# Genome-wide promoter extraction and analysis in human, mouse, and rat

**DOI:** 10.1186/gb-2005-6-8-r72

**Published:** 2005-08-01

**Authors:** Zhenyu Xuan, Fang Zhao, Jinhua Wang, Gengxin Chen, Michael Q Zhang

**Affiliations:** 1 Cold Spring Harbor Laboratory, 1 Bungtown Road, Cold Spring Harbor, NY 11724, USA

## Abstract

Large-scale and high-throughput genomics research needs reliable and comprehensive genome-wide promoter annotation resources. We have conducted a systematic investigation on how to improve mammalian promoter prediction by incorporating both transcript and conservation information. This enabled us to build a better multispecies promoter annotation pipeline and hence to create CSHLmpd (Cold Spring Harbor Laboratory Mammalian Promoter Database) for the biomedical research community, which can act as a starting reference system for more refined functional annotations.

## Abstract 2

An investigation of how to improve mammalian promoter prediction by incorporating both transcript and conservation information leads to the creation of CSHLmpd, a mammalian promoter database.

## Background

Gene transcription is regulated by transcription factors (TFs), binding mostly and specifically to the promoter regions. Recent developments of technologies for studying genome-wide transcriptional regulation include microarray expression and chromatin immunoprecipitation (ChIP). The analysis of data from such high-throughput technologies often requires a large set of promoter sequences. Some existing promoter databases for mammals, such as the Eukaryotic Promoter Database (EPD) [1] and the Database of Transcriptional Start Site (DBTSS) [2], were constructed by collecting experimentally identified promoter regions. The promoter data are, however, very limited in these databases. Computational methods have been developed to predict promoters in genomic sequences, but the performance is far from satisfactory, especially for non-CpG-island-related promoters [3,4]. Although known mRNAs have also been used to map the potential promoter regions [5-8] and genome-wide full-length cDNA sequencing projects have contributed lots of very valuable data [9-11], currently only 47-50% of human and mouse genes (or 21% of rat genes) have reference mRNAs (Table 1). It is therefore highly desirable to build a more comprehensive and accurate promoter dataset for the functional genomic community.

We have integrated sequence conservation with our promoter prediction program FirstEF [12] to improve the accuracy of prediction. FirstEF was developed as an *ab initio *human first-exon prediction program, which is capable of predicting noncoding first exons together with the corresponding promoters. It has been used in conjunction with mRNA/expressed sequence tags (EST) transcript information to produce an initial human promoter annotation pipeline (R. Davuluri and I. Gross, personal communication) because gene transcripts and models can be used to identify promoters with high confidence [13]. At the same time, TWINSCAN [14] and other studies [15] have shown that integrating genomic homology information can increase gene-prediction accuracy by about 10% compared with the use of *ab initio *methods alone, and conserved features in promoters have also been used to improve promoter identification in a small dataset [16]. Here, we set out to test if, and to what degree, integrating homology information from mouse and rat genomes can help to further improve human promoter prediction. We found that homologous sequence comparison can substantially increase the prediction accuracy. This enables us to build an improved multispecies promoter annotation pipeline by extracting known and predicted promoters, and to create a comprehensive mammalian promoter database (CSHLmpd) with on-the-fly analysis tools as a valuable public resource to facilitate future mammalian gene-regulatory network studies. As a convenient operational definition, we refer to 'promoter' in this paper as the genomic region (-700, +300) bp with respect to the transcription start site (TSS).

## Results

We used orthologous genes to detect sequence conservation in promoter regions. To do this, we first identified all genic regions in the genomes on the basis of known and predicted transcripts, then collected all known promoters from present promoter annotations in the public databases and all predicted promoters produced by the original FirstEF. These promoters were then linked to downstream genes (see below). We took known promoters from the human-rodent orthologous genes and observed significant conservation in promoter sequences. We then used this conservation signal to improve *de novo *promoter prediction, and in the end constructed a reference promoter database for each of the three mammalian genomes.

### Human, mouse and rat genes and orthologous gene sets

By aligning all known and predicted transcripts to the latest human, mouse and rat genomes we obtained 34,949, 35,073, 30,679 genes (see Materials and methods), which include 29,360, 25,571 and 22,643 canonical genes (based on RefSeq [17] mRNA and Ensembl [18] prediction) in these genomes, respectively. The orthologous relationship of these canonical genes is defined using EnsMart [19], which is based on similarity analysis of Ensembl transcripts and genes. We obtained 19,179 human-mouse-rat three-species orthologous gene triplets, and 1,967, 1,420 and 2,268 human-mouse, human-rat and mouse-rat two-species orthologous gene pairs respectively. Promoter conservation was studied in these orthologous genes.

### Known promoter collection and promoter prediction in human, mouse and rat genomes

For each species we collected known promoters from EPD and DBTSS. We also collected known promoters from GenBank [20] by keyword search (see Materials and methods), and the promoter regions identified by luciferase assay and ChIP of *TAF250 *and *RNA polymerase II *in the Encyclopedia of DNA Elements (ENCODE) regions. These known promoter sequences were aligned with the genome by BLAT [21] to get the locations of TSSs. The total unique known TSSs in human, mouse and rat are 14,314, 8,141 and 943, respectively [21]. We also predicted 608,057, 449,132 and 427,130 promoters in these genomes separately using FirstEF with default parameter setting. Repeats in the genome were not masked. TSS locations of all known and predicted promoters were compared with the identified gene regions. A TSS is assigned to a gene when it is located in the genic region or upstream of the 5' end of the gene by no more than 5 kb (for RefSeq genes) or 20 kb (for other genes). By doing so, we obtained such 'gene-related' TSSs/promoters for further analysis. Predicted 'gene-related' promoters are also defined as 'transcript-supported promoters' if they overlap the 5' end of any transcript in a gene. Other predicted TSSs that were not gene-related were potential 'novel TSSs' and were not further analyzed. We used known promoters as training data to detect promoter conservation signal and then compared it with the signal in predicted promoters to reduce false-positive promoter predictions.

### Statistical similarity among known promoters of orthologous genes

#### Pairwise comparison of known promoters

**Figure 1:**
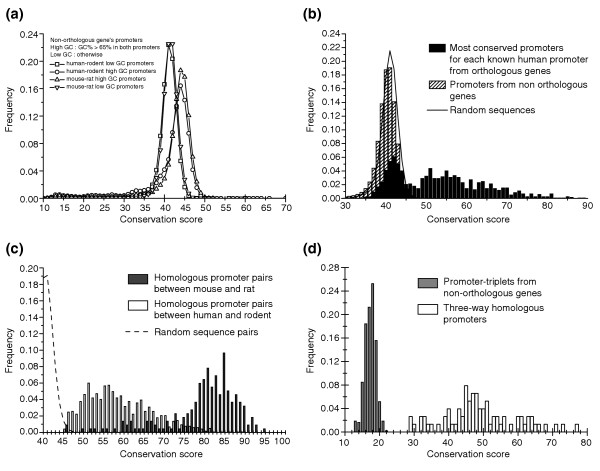
Distribution of conservation scores in promoter alignments. **(a)** Pairwise promoter alignments of human-rodent and mouse-rat non-orthologous genes (control set II) with different promoter GC content. **(b)** Pairwise promoter alignments of most conserved promoter pairs and randomly selected 1 kb sequence pairs (control set I). **(c)** Alignments of mouse-rat and human-rodent homologous promoter pairs. **(d) **Three-way promoter alignments of homologous promoter triplets and sequence triplets from control set II.

Of the orthologous gene pairs, 3,649 human-rodent and 214 mouse-rat pairs have known promoters in both species. We compared these known promoters by ClustalW [22] to measure the conservation in promoters. The conservation score is defined as the percentage of identical base-pairs in a 1 kb region. Using randomly selected known promoters of non-orthologous genes (see Materials and methods), we found that such conservation positively correlates with the GC content, especially when GC content is greater than 65%, and surprisingly, that the conservation distribution is independent of the species used for comparison (Figure 1a). We also measured the conservation for randomly selected 1 kb genomic DNA sequences, and found the same distribution of conservation score (Figure 1b, species-related data are not shown). Therefore, we chose the 99% quantile as the conservation cutoff for discriminating the pairwise 'high-scoring promoters' (that is, 1% error threshold or 1PET). We found that the conservation threshold is 48.8% for sequences of high GC content (greater than 65%), and 45.8% for the rest. The distribution of conservation score in known human-rodent promoter pairs is shown in Figure 1b, which consists of two mixed populations: one is similar to that of the sequence pairs in the two control sets, and the other is peaked much higher than 1PET.

We then defined a promoter pair as a homologous promoter pair, and the promoters as homologous promoters, if the conservation score is higher than 1PET (the pairwise cutoff rule). Using these cutoffs, we found 2,841 of 4,140 human known promoters in those 3,649 human-rodent orthologous gene pairs, and 152 of 229 mouse known promoters in those 214 mouse-rat orthologous gene pairs. In total, around 66-68% of known promoters can match highly conserved counterparts in the orthologous genes. The average conservation score is around 55% between human-rodent homologous promoter pairs, and 85% between mouse-rat homologous promoter pairs (Figure 1c).

#### Three-species promoter comparisons

We also analyzed known promoter conservation in 158 human-mouse-rat three-way orthologous gene triplets, which have 249 all-species promoter triplets. Using ClustalW to randomly align selected 1 kb sequences from human, mouse and rat genomes, we found that only 1% of the 1 kb triplets had conservation score higher than 21.8%. Here, the conservation score is defined as the percentage of identical base-pairs in the multiple alignments of 1 kb sequences. Using this cutoff, we identified 76 known promoter triplets, and the distribution of conservation score is shown in Figure 1d.

In the genome, functional regions (such as coding regions) are usually conserved under selection pressure during evolution. Hence the significantly higher conservation of homologous promoter pairs and triplets encouraged us to test whether it could be used to improve promoter prediction.

### Improving promoter prediction by incorporating both mRNA annotation and promoter conservation information

**Figure 2:**
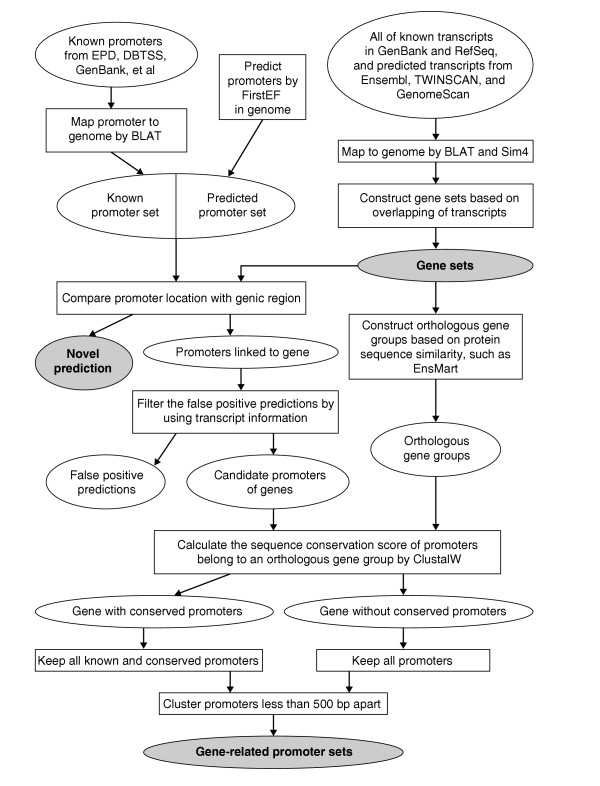
Flowchart of the pipeline to construct the promoter database. Ovals indicate data and rectangles the method. The ovals shaded gray represent the data stored in CSHLmpd.

We are able to combine the conservation signal in homologous promoters with promoter models used in FirstEF program to improve promoter prediction. We compared the performance of four methods. Method 0 is original FirstEF. Method 1 is a *de novo *FirstEF (with the post-clustering filter [23]) that only keeps the best-predicted promoters from the original FirstEF predictions within a 1,000 bp region. Method 2 uses transcript information to filter out the false positives of Method 0 predictions that are located within the gene region. Method 3 incorporates conservation signals into Method 2: first, predicted promoters are selected by using Method 2, and then for genes with homologous promoters, only the conserved predicted promoters will be reported (see Materials and methods and Figure 2). Here the conservation signal was measured between human and rodent promoters in the same orthologous gene pair, and the pairwise cutoff rule defined above was used to identify homologous promoters.

We collected 8,949 well annotated human genes, each of which has at least one known TSS and has at least one orthologous gene in mouse or rat, to do the test. There are in total 13,313 unique known TSSs for these human genes, with 9,806 being at least 500 bp apart (see Materials and methods). In both sets, we shortened each gene by 5 kb (or half of the gene length if the gene is shorter than 5 kb) from its 5' end to simulate 5' incomplete genes that are most common in the current gene annotations.

**Figure 3:**
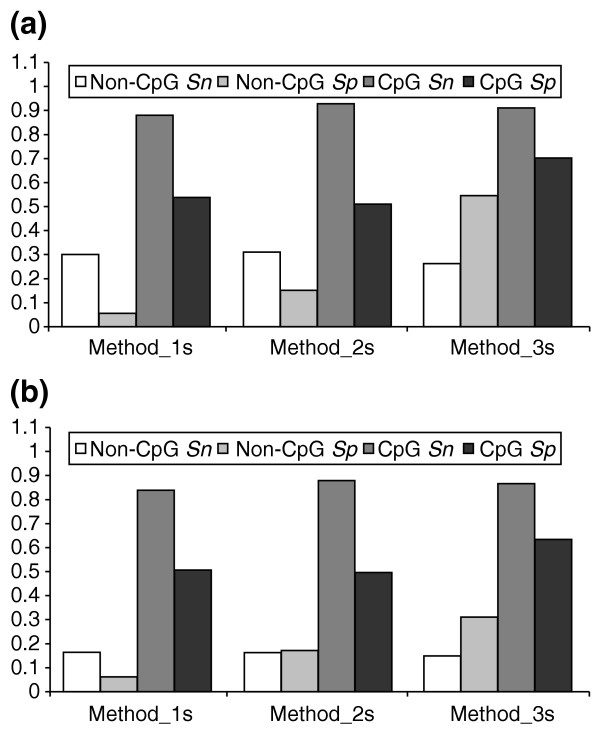
Sensitivity and specificity of promoter prediction for CpG-island related and non-CpG-island related promoters in different gene sets. **(a)** 5,893 human genes with homologous rodent promoters. **(b)** All 8,949 human genes in the test set. The definition of different methods is described in the text and in Materials and methods.

We found that by incorporating mRNA (Method 2) and promoter conservation information (Method 3), we could improve promoter prediction over the *de novo *FirstEF (Method 1) (Table 2). With conservation and mRNA information together, we achieved 66% in specificity and 69% in sensitivity on the 13,313 unique TSS set, corresponding to improvements of 20% and 2% respectively. Comparing this with the original FirstEF prediction (Method 0), we found that although sensitivity dropped 3%, an improvement of 20% in specificity is well worth the effort. Just using transcript information, Method 2 can improve on Method 1 by 11% in specificity and 3% in sensitivity (Table 2a). For those 9,806 known TSSs separated by at least 500 bp, we found that Method 3 still gives the largest improvement, with specificity (*Sp*) and sensitivity of prediction (*Sn*) reaching 60% and 66% (26% and 2% higher than those by Method 1), respectively (Table 2b). Of the 8,949 human genes, 5,893 (66%) have homologous promoters, and the specificity and sensitivity of promoter prediction for these genes by Method 3 are 69% and 82%, respectively (Table 2c). On the basis of the new definition of CpG-island [24], we found that the prediction of CpG-island related promoter has higher sensitivity and specificity (Figure 3a,b), consistent with the fact that FirstEF offers better prediction for CpG-related promoters than non-CpG-related ones. For CpG-island related promoters with homologous counterpart, the *Sp *and *Sn *of the prediction can reach 70% and 91% respectively. Very strikingly, the improvement for non-CpG related promoter prediction by homology information is much more dramatic (Figure 3). These results clearly show the considerable value of cross-species comparison in promoter prediction.

### Incorporation of cross-species conservation in whole-genome promoter/TSS prediction

Encouraged by the enhancement in promoter prediction performance obtained by combining FirstEF promoter models with conservation signal and transcript information, we applied Method 3 to annotate human, mouse and rat genomes (Figure 2). In addition to the known and the original FirstEF-predicted TSSs, we defined two types of surrogate TSSs: bidirectional TSSs and RefSeq END TSSs. If the intergenic region between two adjacent 'head-to-head' (divergent) genes is shorter than 2 kb, their 5' ends are defined as bidirectional TSSs even if no promoter is predicted. For a gene with a RefSeq mRNA, the 5'-end location of the RefSeq mRNA is defined as RefSeq END TSS if there is no other known or predicted TSS linked to this gene. For each gene, we always keep its known promoters and assign these with the highest reliability. Method 3 was then used to select representative promoters from other predicted promoters of this gene, with homologous promoters having higher priority to be chosen (see Materials and methods for details) to reduce the false-positive rate. For simplicity, two TSSs of the same gene are regarded as alternative TSSs. By doing this, we obtained 55,513, 46,207 and 37,479 known and predicted promoters for 26,820, 22,228 and 21,125 genes in human, mouse and rat, respectively. With the current methods, we could not assign promoters for the remaining 8,129, 9,481 and 9,554 human, mouse and rat genes (most of them are predicted genes or only have single EST matches, see below). The detailed statistics are listed in Table 3. After comparing gene boundaries and TSSs to the CpG-islands (see Materials and methods), we found that most RefSeq genes are CpG-island related. In total, 68%, 54% and 56% promoters obtained above for human, mouse and rat are CpG-island related.

From the above promoter/TSS sets, we found 21,594, 21,501 and 17,257 homologous promoters for 13,432, 14,626 and 12,302 genes in human, mouse and rat. Of the mammalian canonical genes with orthologous genes, 60% to 70% have homologous promoters. However, our methods can assign promoters for only a small portion of the TWINSCAN and GenomeScan [25] predicted genes (42%), compared to 82% of the canonical genes (data not shown). This may be due either to the sensitivity of FirstEF, or to the fact that most predicted genes start from putative translational initiation sites (ATG) and the missing 5' exons and intron regions can span beyond our promoter search limit (20 kb upstream of the predicted gene boundary). The lack of complete 5' ends in non-RefSeq genes can also explain why we saw them to be less likely to be CpG-island related.

### Cold Spring Harbor Laboratory Mammalian Promoter Database

To store the information about all the genes and promoters we annotated, we have constructed the Cold Spring Harbor Laboratory Mammalian Promoter Database (CSHLmpd [26]). It consists of three species-specific promoter sub-databases for human (HSPD), mouse (MMPD) and rat (RNPD). They are linked by homologous promoters wherever orthologous gene information is available. Each is currently equipped with two basic front-end components: a genome-wide browser, Gbrowse [27], to display information graphically; and a query-fetch system to query and extract promoters based on a gene identifier (such as GenBank accession number, UniGene [28] cluster ID, LocusLink [28] ID or gene name). In CSHLmpd, users can either search for promoters of their genes of interest in one species or get homologous promoters from other species. To make the database both a data resource and an analysis platform, we provide two sequence-alignment tools for homologous promoter analysis. ClusterW is for global multiple sequence alignment in the regions of user-selected promoters, and PromoterWise, a local alignment tool, is embedded to align each pair of promoter regions (E. Birney, unpublished data). We have also used MLAGAN [29] to do global multiple sequence alignment in the regions that include genes and their 5,000-bp upstream sequences to show the conservation at a larger scale. More promoter-analysis tools will be added in the future.

**Figure 4:**
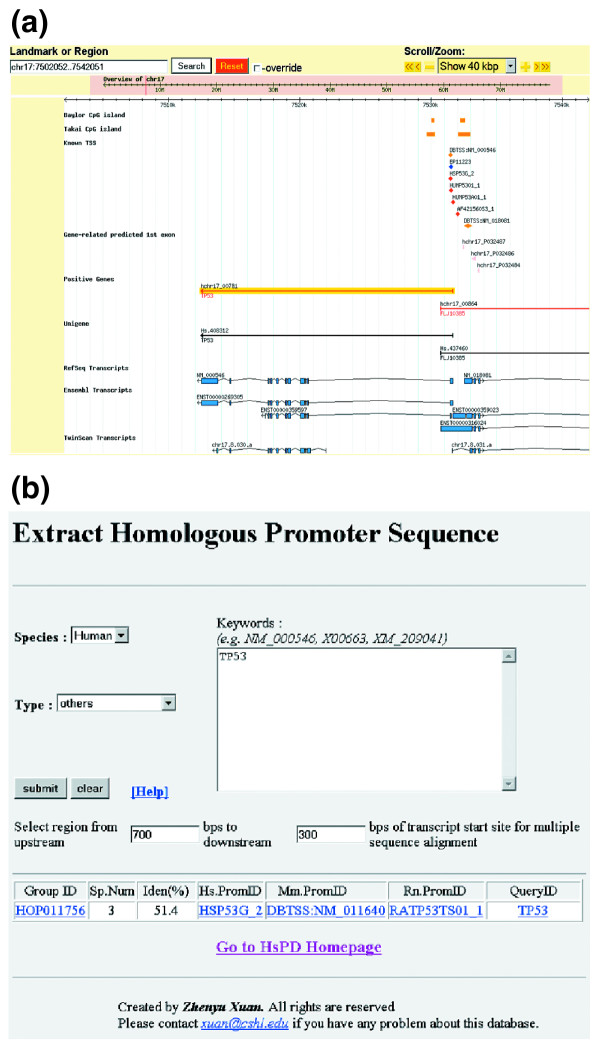
Screen shots of the CSHLmpd user interface. **(a)** Gbrowse for genome-wide gene and promoter display. **(b) **Homologous promoter search and analysis.

In addition, there is another related database, the Transcription Regulatory Element Database (TRED) [30]. It includes curated biological information, such as transcription factor binding sites (TFBSs) and regulation pathways/networks as well as *cis*-element analysis tools. Figure 4 shows some representative screen shots of the database user interface. For the user's convenience, we have classified the promoter quality in the following order (from the highest to the lowest): known promoters (EPD, DBTSS, GenBank annotation, promoters identified by luciferase assay or ChIP), RefSeq END promoters, transcript-supported promoters, bidirectional promoters, and other predicted promoters (see Materials and methods). If promoters with different qualities are linked to a gene, users can choose to retrieve only the most reliable one, any, or all of them. This promoter database is publicly available and all data are free for academic use.

### Facilitating large-scale gene regulation studies and promoter array construction

Expression microarray and ChIP-chip (ChIP followed by microarray analysis of DNA) technologies have become important and widely used approaches to study gene expression and regulation at large scales. Being able to extract a large set of mammalian promoter sequences is a critical step for such studies.

To demonstrate the use of CSHLmpd, we have extracted a promoter sequence dataset for the Affymetrix human array HG-U133A. Out of the total of 22,283 probe sets for most known human genes [31] on this array, from the annotation we were able to obtain promoters from CSHLmpd for 20,903 of them. Because multiple probe sets can belong to the same gene, 13,014 promoters were retrieved. These include 6,052 known promoters and 4,550 predicted homologous promoters. No promoter could be assigned for only 1,380 probe sets. Among these, 448 were mapped to 353 genes without promoter information in our database, and 932 were created from poorly aligned mRNAs and ESTs, which were not used to construct the genes in the first place, or from other ESTs that do not overlap with any gene in our database (see Materials and methods). This HG-U133A Affymetrix promoter set can be freely downloaded from our FTP server [32], where one can also find separately prepared promoter sequence sets for all human, mouse and rat RefSeq genes. These RefSeq gene promoter sets include all DBTSS-defined promoters and RefSeq END TSS. Users can also create other customized promoter sequence sets for different arrays (or gene indices) using the CSHLmpd query tools. We also plan to provide more customized promoter sequence sets for making promoter chips that can be used for large-scale ChIP-chip studies or epigenetic mapping projects (such as for DNA methylation).

## Discussion

Our method first collected known and predicted promoters in the whole genome. Then transcript and conservation information were used to filter the false positives from the predictions. Our test presented in this paper has proved that using both transcript and conservation information, together with FirstEF, will improve the accuracy of promoter prediction compared with the use of transcript information alone (for example, PromSer, Source). To our knowledge, this is the first attempt to integrate conservation information with *de novo *first-exon prediction on a genome-wide scale.

In collaboration with an experimental group (L. Stubbs, personal communication), we previously tested our FirstEF prediction on 48 human genes in chromosome 19 using reporter assays. Among these, 26 genes had promoters correctly predicted, and eight did not. This gave a sensitivity and specificity of 54% and 65%, respectively, at the gene level. However, there were a total of 105 predicted promoters around these genes, which led to a specificity of only 25% at the promoter level (data not shown). Therefore, while the experimental evaluation proves that *de novo *FirstEF performs well in predicting promoters for novel genes, it also shows its limitations on prediction specificity. A more systematic experimental test of 300 mouse promoters will be found in [33]. Our work presented here shows that both mRNA information and cross-species conservation can significantly improve the specificity of promoter prediction.

We have also demonstrated that conservation signal can be integrated with promoter models to improve the accuracy of promoter prediction. Our method uses conservation signal in the potential promoter regions, which can greatly reduce false positives when comparing using just mRNA or conservation information alone, especially when known mRNAs only have partial coding regions. Furthermore, without mRNA information, homologous information by itself cannot produce better overall prediction (data not shown), partly as because of a higher degree of conservation in exons. To decrease false predictions caused by exon conservation as much as possible, we not only used the information from known genes, but also predicted genes from some well known gene-finding methods. In this way, we can reduce the promoter search regions for known genes, and may obtain additional theoretical evidence for predicted genes when their promoters are predicted [4]. These potential novel genes with predicted promoters, especially when the promoters are evolutionarily conserved, could be valuable candidates for experimental validation. In our recent experiments, we have shown that about 25% of those novel genes have spliced transcripts [33].

To detect the conservation in promoter regions, we tested several different promoter definitions. They included upstream 200 bp of TSSs, -400 to +100 bp, -700 to +300 bp, and -1,500 to +500 bp around TSS. We found that the peak of the conservation score is closer to that of the control sequence set when promoter regions are too short or too long. Among these four promoter definitions, -700 to +300 bp around TSSs gave the best discrimination between the known promoter-training set and the control set. This indicated that many conserved TFBSs tend to cluster in the approximately 1 kb region near the TSS [34].

In our studies, we have observed that, if lower thresholds of the original FirstEF (such as P_exon _= 0.3, P_promoter _= 0.25, P_donor _= 0.25) are used, the prediction sensitivity can be increased at the expense of specificity. In this case, however, even though mRNA and conservation information could help regain some specificity, the overall accuracy would actually be worse than that with default FirstEF thresholds (data not shown).

We cannot identify conservation signal for 27% of known human promoters and 17% of known rodent promoters (see our FTP site [32]). This may be due to the faster promoter divergence in the corresponding genes. The percentage of predicted promoters without homology that were detected was higher than that of known promoters because of the bias of existing known promoter data and false positives of promoter prediction. We hope to develop more sensitive methods for promoter-specific conservation detection in order to improve promoter prediction in the future.

## Materials and methods

### Human, mouse and rat genome releases

Human NCBI build 35 (May 2004), mouse mm5 (May 2004), and rat assembly rn3 (June 2003), were downloaded from the University of California at Santa Cruz (UCSC) website [35].

### Genic region identification in the genomes

mRNAs from RefSeq and GenBank (mRNA), and transcripts predicted by Ensembl, TWINSCAN and GenomeScan (RefSeq XM) in the annotation of UCSC genome assemblies were obtained. They were aligned to the genomes by BLAT and Sim4 [36] programs. Transcripts with more than 10% nucleotides unaligned or with less than 95% identity in the aligned regions were excluded. Transcripts were regarded as overlapping if their exons shared at least 1 bp, and a genic region was defined as a continuous genomic DNA region that covers all overlapped transcripts. Gene type was based on the most reliable transcript for this gene, and the order of transcript reliability is: RefSeq > mRNA > Ensembl > RefSeq XM > TWINSCAN. All ESTs were also mapped to the genomes in the same way. ESTs that overlap an identified genic region were included as transcripts of this gene without changing the genic region boundary. The UniGene ID was linked to the gene on the basis of its transcripts. For genes with Ensembl transcript ID, using the information from Ensembl's EnsMart, we marked the orthologous gene sets in our identified genes.

### Known promoter collection

All promoter sequences in EPD (release 74) and DBTSS (release 2.0) were extracted. Promoter information and sequences were also retrieved from GenBank (dated 21 February 2003) using 'exon number = 1', 'prim_transcript', 'precursor_mRNA', and 'promoter' as keywords. The promoter regions identified by luciferase assay and ChIP of *TAF250 *and *RNA polymerase II *in the ENCODE regions were obtained from the UCSC genome browser and included. All sequences were mapped to the genomes by BLAT to obtain their locations of TSSs. Two identical TSSs were regarded as one unique TSS.

### Whole-genome promoter prediction

With default thresholds (P_exon _= 0.5, P_promoter _= 0.4, P_donor _= 0.4), original FirstEF was run on each chromosome of the three genomes without repeat masking, and the output was filtered by different methods described below. Predicted and known TSSs were linked to the closest gene if they were located either in the gene region or in the 20 kb upstream of the gene (if the gene has RefSeq mRNA, the distance was limited to 5 kb), and these promoters/TSSs were collected as 'gene-related promoters/TSSs'. Predicted promoters overlapping the 5' end of any transcript in a gene are defined as 'transcript-supported promoters'.

### Conservation in control sets

Regions of 1,000 bp were randomly extracted from the genome of each species to make sequence pairs or triplets. Control set I included 1 million such sequence pairs for every two species, and 1 million triplets for the three species. We also selected genes from different species that are not orthologs, and randomly picked promoters belonging to these genes to make 1 million promoter pairs and 1 million triplets for control set II. One million high-GC content (>65%) pseudo promoter pairs were also selected. ClustalW was used to carry out multiple sequence global alignment for each pair or triplet with the conservation score defined as the ratio of identical base-pairs divided by 1,000.

### Calculation of conservation for known promoters in orthologous genes

For genes with known TSSs, we extracted (-700, +300) bp regions with respect to the TSSs from the genomes as promoter sequences. We aligned each promoter of a gene in one species with each of the known promoters of its orthologous genes by ClustalW and calculated the conservation scores. The maximum score of all these promoter pairs or triplets was used to describe the conservation of this promoter.

### CpG island relationship

We used the new CpG-island definition [24] to search genomes of the three species to collect CpG islands. A gene is considered as CpG-island-related only if there is at least one CpG island overlapping the region of (-2,000 to around +500) bp at its 5' end. A TSS/promoter is considered as CpG-island-related if at least one CpG island can overlap the region of (-2,000, +500) bp with respect to the TSS.

### Post-clustering script for selecting promoters at least 500 bp apart

For all the gene-related promoters, we first ordered the known ones on the basis of the distance between TSSs defined in the promoters to the gene 5' end defined by mapped transcripts. The promoters with shorter distances were then selected, and the rest were compared to the selected ones. Only those that were separated by at least 500 bp from any of the selected promoters were kept. The same selection procedure was used for homologous promoters, transcript-supported promoters and other promoters. As a result of such post-clustering, all the selected promoters of a gene were separated by at least 500 bp.

### Evaluation of promoter prediction by simulation

The test set comprised 8,949 genes with 13,313 known TSSs. To simulate the 'partial genes' that often exist in the databases, we truncated each identified genic region by 5 kb (or half of the gene length if the gene is shorter than 5 kb) at the 5' end, including the parts of cDNAs that extend into this region. On the basis of such new gene boundaries, we reselected all gene-related promoters from the predictions by original FirstEF (Method 0). Each promoter was compared with promoters of the orthologous genes (if available) by ClustalW to calculate the conservation score, and they were defined as the homologous promoters if the conservation score obeyed the pairwise or three-way cutoff rules.

*De novo *FirstEF (Method 1) selected the best-predicted promoters (with the highest probability in the promoter region) from the original FirstEF predictions in a 1,000 bp region. Method 2 compared RNAs or predicted transcripts with original FirstEF predictions that were gene-related to filter out predicted promoters that were neither located in the upstream of the genic region nor transcript-supported, and Method 3 first used Method 2 to select promoters, and then for a gene with homologous promoters, only those homologous promoters were selected as output for the gene (see also Figure 2). Post-clustering was used in promoter selection from the output of Method 1, Method 2 and Method 3 for tests in the 9,806 known TSSs of 500 bp apart, and such combined methods were called Method 1s, Method 2s, and Method 3s respectively. A predicted TSS was regarded as a 'correct TSS' if its distance to a known TSS was shorter than 500 bp, and this known TSS was regarded as 'correctly predicted' simultaneously. The sensitivity of prediction (*Sn*) was defined as the ratio between the numbers of correctly predicted and known TSSs used in the validation. Specificity (*Sp*) was the number of correct TSSs divided by the total number of predicted promoters.

### Cold Spring Harbor Laboratory mammalian promoter database construction

We first collected all gene-related TSSs in human, mouse and rat genomes. For genes with RefSeq mRNAs but no known or predicted promoters, the 5' ends of the RefSeq sequences were considered as their TSSs and called Refseq ND TSS. They were also defined as transcript-supported. For two adjacent divergent genes with their 5' ends less than 2 kb apart, we defined their 5' gene boundaries as 'bidirectional TSSs' if no other type of TSS could be found in the intergenic region between them. All promoters of the orthologous genes were aligned by ClustalW to find homologous promoters in the same way as done in the evaluation step. Method 3s was used to select the final promoter set. Known promoters filtered out by the post-clustering script were also included in the database after the selection to make the known promoter data as complete as possible. All these selected promoters were stored in a MySQL database. Gene features contained in the database include genome location, overlapping transcripts, UniGene ID, LocusLink ID, and gene name if available. Promoter features included TSS location, first donor and acceptor sites if available, corresponding gene, overlapped transcript for a transcript-supported promoter, and promoter type. Promoter type refers to the source type, which was also used to represent their reliability in the order of: known promoters (EPD, DBTSS, GenBank annotation, promoters identified by luciferase assay or ChIP), RefSeq END promoters, promoters of divergent genes (bidirectional TSS), transcript-supported promoters, as well as other gene-related promoters that were predicted. Homologous promoters were also marked. In addition to gene-related promoters, all other predicted promoters located in the intergenic regions were included in the database. They were regarded as predicted novel promoters and were of the lowest reliability.

### Promoter set for the Affymetrix microarray

For each probe set in the gene chip, its gene index and/or chromosome location information were used to find the corresponding gene in our promoter database. The most reliable promoter of this gene was reported for this probe set. If no gene could be assigned to a probe set, the closest predicted novel promoter in its upstream region was taken if the distance between the promoter and probe set was less than 20 kb.

### Data availability

All 8,949 human genes and 13,313 human known promoters used in the test can be downloaded from our FTP site at [37], the promoter set for Affymetrix array HG-U133A is in [38], the promoter set of all RefSeq genes is in [39], all known promoters in CSHLmpd can be downloaded from [40].

## Figures and Tables

**Table 1:** Number of genes and transcripts of different types in the three mammalian genomes

Type	HSPD		MMPD		RNPD	
	Gene*	Transcript^†^	Gene	Transcript	Gene	Transcript

RefSeq	17,354	22,425	16,329	17,438	6,400	6,807
mRNA	8,846	106,279	2,641	40,552	1,967	11,116
Ensembl	3,160	33,653	6,601	31,022	14,276	27,989
RefSeq_XM	2,400	6,105	4,974	5,829	3,021	15,023
TwinScan	3,189	25,633	4,528	25,583	5,015	25,499
EST	0	4,488,530	0	3,254,853	0	477,321
Total	34,949	4,682,625	35,073	3,375,277	30,679	563,755

*Number of genes in non-overlapping gene types. ^†^Number of all transcripts of this type.

**Table 2:** Sensitivity and specificity of promoter prediction with different methods

	*Sn*	*Sp*
**(a) **13,313 unique TSSs in 8,949 human genes		
Method 0*	72%	46%
Method 1^†^	67%	46%
Method 2^‡^	70%	57%
Method 3^§^	69%	66%
		
**(b) **9,806 TSSs of 500 bp apart in 8,949 human genes		
Method 1 + script^¶^	64%	33%
Method 2 + script	67%	44%
Method 3 + script	66%	60%
		
**(c)** 6,356 TSSs of 500 bp apart in 5,893 human genes with homologous promoters		
Method 1 + script	80%	37%
Method 2 + script	84%	46%
Method 3 + script	82%	69%

*Method 0 used *original FirstEF *alone to predict promoters in the upstream and genic regions of these genes. ^†^Method 1 used *de novo FirstEF *to predict promoters in the upstream and genic regions of these genes. ^‡^Method 2 compared mRNAs or predicted transcripts with *original FirstEF *predictions to filter out promoters that were neither located in the upstream of the gene region nor overlapping with the 5'-end of any transcripts of this gene. ^§^Method 3 tried to first find the promoters in one gene that have homologous rodent promoters. If no such promoters were found, it used Method 2 to select promoters for this gene. ^¶^script, a post-clustering script to select representative TSSs from the output of each method described above that were at least 500 bp apart (see Materials and methods for details).

**Table 3:** Statistics of promoters and genes in CSHLmpd

	HSPD	MMPD	RNPD
Total genes	34,949	35,073	30,679
Known genes (RefSeq and mRNA)	26,200	18,970	8,367
Canonical genes (RefSeq, mRNA, and Ensembl)	29,360	25,571	22,643
Genes with promoters	26,820	25,592	21,125
Genes with homologous promoters	13,432	14,626	12,302
Predicted genes with promoters	4,340	7,343	13,230
Total promoters*	55,513	46,207	37,479
Known promoters	14,314	841	943
FirstEF predicted promoters	39,233	34,994	34,227
Transcript-supported FirstEF predicted promoters	19,331	16,913	11,798
RefSeq END promoters	1,828	2,988	2,270
Bidirectional gene promoters	138	84	39
Core promoters	26,820	25,592	21,125
Homologous promoters	21,594	21,501	17,257
Homologous known promoters	10,561	6,854	817
			
CpG-island related RefSeq genes	12,259 (71%)	9,831 (60%)	2,987 (47%)
CpG-island related other mRNA genes	2,679 (30%)	993 (38%)	907 (46%)
CpG-island related canonical genes	15,707 (54%)	12,293 (48%)	8,420 (37%)
CpG-island related promoters	37,572 (68%)	24,726 (54%)	20,826 (56%)
CpG-island related known promoters	10,332 (72%)	5,115 (63%)	444 (47%)
CpG-island related predicted promoters	26,936 (69%)	19,363 (55%)	20,207 (59%)
CpG-island related RefSeq END promoters	187 (10%)	201 (7%)	153 (7%)
CpG-island related bidirectional gene promoters	53 (38%)	47 (56%)	22 (56%)
CpG-island related homologous promoters	13,974 (82%)	11,867 (76%)	9,372 (80%)

*Predicted promoters were separated with other predicted or known promoters by at least 500 bp.
